# Racial and Ethnic Disparities in the Use of Prostate Magnetic Resonance Imaging Following an Elevated Prostate-Specific Antigen Test

**DOI:** 10.1001/jamanetworkopen.2021.32388

**Published:** 2021-11-08

**Authors:** Nino Abashidze, Chad Stecher, Andrew B. Rosenkrantz, Richard Duszak, Danny R. Hughes

**Affiliations:** 1 Haub School of Environment and Natural Resources, University of Wyoming, Laramie; 2 College of Health Solutions, Arizona State University, Phoenix; 3 Department of Radiology, NYU Langone Medical Center, New York, New York; 4 Department of Radiology and Imaging Sciences, Emory University, Atlanta, Georgia; 5 School of Economics, Georgia Institute of Technology, Atlanta

## Abstract

**Importance:**

Prostate cancer screening and diagnosis exhibit known racial and ethnic disparities. Whether these disparities persist in prostate magnetic resonance imaging (MRI) utilization after elevated prostate-specific antigen (PSA) results is poorly understood.

**Objective:**

To assess potential racial and ethnic disparities in prostate MRI utilization following elevated PSA results.

**Design, Setting, and Participants:**

This cohort study of 794 809 insured US men was drawn from deidentified medical claims between January 2011 and December 2017 obtained from a commercial claims database. Eligible participants were aged 40 years and older and received a single PSA result and no prior PSA screening or prostate MRI claims. Analysis was performed in January 2021.

**Main Outcomes and Measures:**

Multivariable logistic regression was used to examine associations between elevated PSA results and follow-up prostate MRI. For patients receiving prostate MRI, multivariable regressions were estimated for the time between PSA and subsequent prostate MRI. PSA thresholds explored included PSA levels above 2.5 ng/mL, 4 ng/mL, and 10 ng/mL. Analyses were stratified by race, ethnicity, and age.

**Results:**

Of 794 809 participants, 51 500 (6.5%) had PSA levels above 4 ng/mL; of these, 1524 (3.0%) underwent prostate MRI within 180 days. In this sample, mean (SD) age was 59.8 (11.3) years (range 40-89 years); 31 350 (3.9%) were Asian, 75 935 (9.6%) were Black, 107 956 (13.6%) were Hispanic, and 455 214 (57.3%) were White. Compared with White patients, Black patients with PSA levels above 4 ng/mL and 10 ng/mL were 24.1% (odds ratio [OR], 0.78; 95% CI, 0.65-0.89) and 35.0% (OR, 0.65; 95% CI, 0.50-0.85) less likely to undergo subsequent prostate MRI, respectively. Asian patients with PSA levels higher than 4 ng/mL (OR, 0.76; 95% CI, 0.58-0.99) and Hispanic patients with PSA levels above 10 ng/mL (OR, 0.77; 95% CI, 0.59-0.99) were also less likely to undergo subsequent prostate MRI compared with White patients. Black patients between ages 65 and 74 years with PSA above 4 ng/mL and 10 ng/mL were 23.6% (OR, 0.76; 95% CI, 0.64-0.91) and 43.9% (OR, 0.56; 95% CI, 0.35-0.91) less likely to undergo MRI, respectively. Race and ethnicity were not significantly associated with mean time between PSA and MRI.

**Conclusions and Relevance:**

Among men with elevated PSA results, racial and ethnic disparities were evident in subsequent prostate MRI utilization and were more pronounced at higher PSA thresholds. Further research is needed to better understand and mitigate physician decision-making biases and other potential sources of disparities in prostate cancer diagnosis and management.

## Key Points

**Question:**

Are there racial and ethnic disparities in patients undergoing prostate magnetic resonance imaging after receiving an elevated prostate-specific antigen test result?

**Findings:**

In this cohort study of 794 809 commercially insured men from 2011 to 2017 undergoing a prostate-specific antigen test, Black and Hispanic patients were significantly less likely than White patients to undergo prostate magnetic resonance imaging after receiving an elevated test score.

**Meaning:**

These results suggest that racial and ethnic disparities exist in the use of subsequent prostate magnetic resonance imaging, highlighting a need to better understand and mitigate physician decision-making biases and other potential sources of the disparities in the identification and management of prostate cancer.

## Teaser

This cohort study examines disparities based on race and ethnicity in prostate cancer screenings following elevated prostate-specific antigen levels among US men.

## Introduction

In the US, racial and ethnic disparities have been identified in multiple aspects of prostate cancer diagnosis and treatment.^1,2,3,4^ Specifically, Black patients are less likely to undergo appropriate prostate-specific antigen (PSA) screening,^5^ less likely to undergo intensive follow-up while on close monitoring for low-risk prostate cancer (ie, active surveillance),^6,7^ and less likely to undergo PSA surveillance after radical prostatectomy.^8^ Such variation in care contributes to increased mortality in Black patients diagnosed with prostate cancer.^4,9^

Accurate prostate cancer diagnoses can help reduce disparities in health outcomes. Traditionally, men are screened for prostate cancer by PSA, and those with elevated PSA results then undergo prostate biopsy.^10^ However, systematic nontargeted biopsies both miss clinically meaningful prostate cancer and overdiagnose insignificant prostate cancer.^11,12^ Recently, prostate magnetic resonance imaging (MRI) has emerged as a means to evaluate men with an elevated PSA before possible prostate biopsy^13,14,15,16^ and its use is rapidly increasing nationally.^17^ Prostate MRI may identify suspicious regions in the prostate to target for subsequent biopsy, significantly increasing the likelihood of identifying clinically meaningful prostate cancer,^18^ and prostate cancer detection by MRI-targeted biopsy is not significantly different between Black vs White patients.^19^ Additionally, a prostate MRI may obviate the need for biopsy, thus decreasing the overdiagnosis of insignificant prostate cancer.^20^

The American Urological Society (AUA) and National Comprehensive Cancer Network have both recognized the important role of prostate MRI for prostate cancer diagnosis.^21,22^ However, consensus is lacking regarding the use of MRI in the initial detection of prostate cancer, and accepted guidelines are not yet available. This lack of standardization likely fosters both variation and disparities in prostate MRI utilization. A 2018 study,^23^ for example, showed variation in the utilization of prostate MRI among Medicare beneficiaries. For this study, we used a large commercial and Medicare Advantage health insurance claims database combined with data on PSA test results to examine racial and ethnic disparities in the use of prostate MRI following receipt of elevated PSA values.

## Methods

This cohort study used deidentified data from an administrative database containing health insurance claims for members of large commercial and Medicare Advantage health plans (Optum). Claims data covered a geographically diverse population, including all 50 states, and additionally contained laboratory test results for approximately 30% of the patients in our sample. The Georgia Institute of Technology’s institutional review board deemed this retrospective study of deidentified administrative claims data as not constituting human subjects research and thus not subject to review. The data analysis was performed in January 2021, and the study followed Strengthening the Reporting of Observational Studies in Epidemiology (STROBE) reporting guideline for reporting cohort studies.

Our study cohort was constructed by first restricting plan members to male enrollees between 2011 and 2017. In May 2013, the AUA published an updated guideline that generally recommends starting PSA screening at age 55. The AUA additionally recommends an individualized approach to PSA screening after age 40 years for higher risk men, such as Black patients, who have been reported to be diagnosed with prostate cancer at younger ages than patients of other races or ethnicities, and recommends against routine PSA screening in men younger than 40 years.^24^ Following the AUA guideline, the study cohort was restricted to patients aged 40 years or older for this analysis. Our sample was additionally restricted to plan members undergoing a single annual PSA test (Prostate Specific Ag, serum; *Current Procedural Terminology* [*CPT*] code, 84153), with no PSA test or prostate MRI claims in previous years and no subsequent PSA test within the study period. These restrictions allowed us to minimize the chances that the included PSA test event was related to prior prostate-related medical conditions. Since no specific *CPT* code exists for prostate MRI, we followed established precedent and defined prostate MRI as pelvic MRI reported with a relevant prostate indication code^23^ (*CPT*, *International Classification of Diseases, Ninth Revision *(*ICD-9*), and *ICD-10* codes are reported in eTable 1 in the Supplement).

### Statistical Analysis

Bivariate and multivariable logistic regression analyses were conducted to estimate associations between PSA test results and subsequent prostate MRI. The outcome variable was a binary indicator of whether a patient underwent a prostate MRI within 180 days after the PSA test date. The treatment variable was a binary indicator of whether a patient had an elevated PSA test result. While no clear consensus exists for the PSA test threshold that determines the need to further evaluate a patient for prostate cancer, studies have revealed several potential PSA levels that suggest the need for further screening. Three PSA levels were explored as a threshold for elevated PSA: (1) PSA level exceeding 4 ng/mL, historically recognized as an appropriate threshold to recommend prostate biopsy^25,26^; (2) PSA level exceeding 2.5 ng/mL, a more recently recognized lower threshold for early detection of prostate cancer^25,26,27^ (patients with PSA results between 2.5 and 4 ng/mL had similar prostate cancer detection rates as patients with PSA between 4 and 10 ng/mL^27^), and (3) PSA threshold exceeding 10 ng/mL, a higher threshold previously associated with high rates of prostate biopsy and high rates of prostate cancer.^28^

Multivariable logistic regression analyses were adjusted for potential confounders including patient age group (ie, ages 40-54, 55-64, 65-74, 75-84, and ≥85 years old), race and ethnicity (Asian, Black, Hispanic, White, and other), whether a patient had Medicare or commercial insurance, type of health plan (HMO or other), PSA test year, and patients’ state of residence. Race and ethnicity were determined by the database as previously documented.^29^ We combined responses of unknown race and ethnicity and missing responses into a single other category. We estimated heteroskedasticity-robust standard errors that were clustered at the state level. Additional regression results were estimated separately for each age group and PSA category.

In addition, Cox proportional hazard models were used to explore associations between PSA test results and the number of days until subsequent prostate MRI. For these models, the outcome variable was the number of days between the PSA test date and a subsequent prostate MRI date (bounded to 180 days), and the models included the same patient-level control variables described above. Cox regressions were also separately estimated for each age group and PSA threshold.

Cohort construction was performed using SAS version 9.4 (SAS Institute). All descriptive and regression analyses were performed using Stata 16 (StataCorp). Statistical tests of significance were assessed with 2-sided tests at the *P* < .05 significance level.

## Results

Between 2011 and 2017, we identified 1 563 534 PSA tests that met our study inclusion criteria. PSA laboratory results were available for 794 809 (50.8%) unique participants of these test events. The mean (SD) age of patients in this sample with complete PSA test results was 59.8 (11.3) years (range 40-89 years). A total of 75 935 (9.6%) of patients were Black, 107 956 (13.6%) were Hispanic, 31 350 (3.9%) were Asian, 455 214 (57.3%) were White, and 124 354 (15.6%) were other race. Additionally, 286 223 (36.0%) of patients were between ages 40 and 54 years old, 224 468 (28.2%) between 55 and 64 years, 197 269 (24.8%) between 65 and 74 years, 71 541 (9.0%) between 75 and 84 years, and 15 308 (1.9%) aged 85 years or older.

Of all available PSA results, 51 500 (6.5%) were above 4 ng/mL and 1524 (3.0%) of these patients underwent a prostate MRI in the subsequent 180 days. Table 1 outlines the annual frequencies and percentages of patients with PSA tests, patients undergoing prostate MRI performed within 180 days, patients with a PSA result above 4 ng/mL, patients with a PSA result above 4 ng/mL undergoing prostate MRI performed within 180 days, and the mean number of days between PSA and subsequent MRI (bounded to 180 days) among patients with a PSA result above 4 ng/mL. Overall, an upward annual trend was observed in the percentage of patients with elevated PSA results undergoing prostate MRI, while the mean number of days between PSA test and prostate MRI fell slightly over this period from 80 days in 2011 to 60 days in 2017. Table 2 reports annual age-stratified frequencies and percentages of patients with PSA tests, patients with a subsequent MRI, patients with PSA levels above 4 ng/mL, patients with a PSA result above 4 ng/mL undergoing prostate MRI, and the mean number of days between PSA test and subsequent MRI among patients with PSA levels above 4 ng/mL. Of note, in 2011, a PSA level above 4 ng/mL was found in 419 patients (1.1%) between ages 40 and 54 years, 1178 (4.4%) between 55 and 64 years, 1298 (9.4%) between 65 and 74 years, 1012 (17.7%) between 75 and 84 years, and 102 (22.0%) of those 85 years and older; similar rates by age group were observed over time.

**Table 1.** Annual Frequency of PSA Testing and Subsequent MRI

Year	Total patients receiving a PSA test, No.	Patients, No. (%)			Mean time between high PSA result and MRI, d^a^
		Undergoing MRI within 180 d	With high PSA result^a^	Undergoing MRI <180 d after high PSA result^a^	
2011	85 589	74 (0.1)	4009 (4.7)	49 (1.2)	80 (43.0)
2012	76 146	94 (0.1)	4110 (5.4)	70 (1.7)	86 (45.5)
2013	89 028	120 (0.1)	5170 (5.8)	84 (1.6)	88 (46.0)
2014	77 786	121 (0.2)	4741 (6.1)	92 (1.9)	70 (46.4)
2015	100 351	247 (0.2)	6191 (6.2)	190 (3.1)	73 (43.5)
2016	130 724	405 (0.3)	8368 (6.4)	321 (3.8)	74 (43.4)
2017	235 185	907 (0.4)	18 911 (8.0)	718 (3.8)	60 (42.8)
Total	794 809	1968 (0.2)	51 500 (6.5)	1524 (3.0)	76 (44.4)

Abbreviations: MRI, magnetic resonance imaging; PSA, prostate-specific antigen.

^a^ High PSA results were considered levels 4 ng/mL or above.

**Table 2.** Annual Age-Stratified Numbers of PSA, Elevated PSA, and MRI Examinations Performed Within 180 Days After a PSA Test

Year	Age, y	Patients, No. (%)				Mean time between high PSA result and MRI, d^a^
		Receiving a PSA test	Undergoing MRI ≤180 d	With high PSA result^a^	Undergoing MRI ≤180 d with high PSA result^a^	
2011	40-54	39 049 (45.6)	13 (0.03)	419 (1.1)	8 (1.9)	81 (35.1)
	55-64	26 498 (31.0)	30 (0.1)	1178 (4.4)	23 (2.0)	71 (41.6)
	65-74	13 859 (16.2)	23 (0.2)	1298 (9.4)	13 (1.0)	101 (42.8)
	75-84	5720 (6.7)	8 (0.1)	1012 (17.7)	5 (0.5)	67 (54.0)
	≥85	463 (0.5)	0	102 (22.0)	0	NA
2012	40-54	33 563 (44.1)	12 (0.04)	366 (1.1)	6 (1.6)	91 (48.7)
	55-64	21 270 (27.9)	31 (0.1)	1002 (4.7)	28 (2.8)	93 (42.9)
	65-74	14 623 (19.2)	36 (0.2)	1530 (10.5)	27 (1.8)	83 (51.5)
	75-84	6214 (8.2)	14 (0.2)	1104 (17.8)	9 (0.8)	70 (32.1)
	≥85	476 (0.6)	1 (0.2)	108 (22.7)	0	NA
2013	40-54	35 775 (40.2)	13 (0.04)	384 (1.1)	9 (2.3)	101 (54.4)
	55-64	24 708 (27.8)	41 (0.2)	1190 (4.8)	34 (2.9)	84 (41.4)
	65-74	19 995 (22.5)	57 (0.3)	2046 (10.2)	37 (1.8)	91 (48.0)
	75-84	6796 (7.6)	8 (0.1)	1112 (16.4)	3 (0.3)	97 (38.8)
	≥85	1754 (2.0)	1 (0.1)	438 (25.0)	1 (0.2)	8 (NA)
2014	40-54	31 320 (40.3)	15 (0.05)	340 (1.1)	8 (2.4)	86 (39.6)
	55-64	21 550 (27.7)	38 (0.2)	1016 (4.7)	26 (2.6)	59 (40.7)
	65-74	16 801 (21.6)	45 (0.3)	1816 (10.8)	39 (2.1)	74 (51.5)
	75-84	6473 (8.3)	21 (0.3)	1161 (17.9)	17 (1.5)	62 (42.5)
	≥85	1642 (2.1)	2 (0.1)	408 (24.8)	2 (0.5)	128 (7.1)
2015	40-54	38 620 (38.5)	31 (0.1)	434 (1.1)	20 (4.6)	75 (40.6)
	55-64	30 049 (29.9)	82 (0.3)	1588 (5.3)	68 (4.3)	77 (44.9)
	65-74	21 064 (21.0)	100 (0.5)	2257 (10.7)	76 (3.4)	74 (45.3)
	75-84	8204 (8.2)	31 (0.4)	1358 (16.6)	23 (1.7)	59 (36.3)
	≥85	2414 (2.4)	3 (0.1)	554 (22.9)	3 (0.5)	52 (23.3)
2016	40-54	46 332 (35.4)	53 (0.1)	546 (1.2)	39 (7.1)	76 (42.6)
	55-64	40 950 (31.3)	121 (0.3)	2137 (5.2)	98 (4.6)	74 (44.4)
	65-74	30 305 (23.2)	181 (0.6)	3288 (10.8)	147 (4.5)	76 (42.6)
	75-84	10 695 (8.2)	46 (0.4)	1779 (16.6)	34 (1.9)	66 (45.5)
	≥85	2442 (1.9)	4 (0.2)	618 (25.3)	3 (0.5)	72 (51.2)
2017	40-54	61 564 (26.2)	66 (0.1)	864 (1.4)	48 (5.6)	61 (39.9)
	55-64	59 443 (25.3)	217 (0.4)	3314 (5.6)	164 (4.9)	61 (42.6)
	65-74	80 622 (34.3)	458 (0.6)	8809 (10.9)	377 (4.3)	60 (42.6)
	75-84	27 439 (11.7)	155 (0.6)	4527 (16.5)	121 (2.7)	57 (44.9)
	≥85	6117 (2.6)	11 (0.2)	1397 (22.8)	8 (0.6)	88 (35.9)

Abbreviations: MRI, magnetic resonance imaging; NA, not applicable; PSA, prostate-specific antigen.

^a^ High PSA results were considered levels 4 ng/mL or above.

Annual frequencies and percentages of patients with PSA tests stratified by race and ethnicity, patients undergoing prostate MRI, patients with a PSA level above 4 ng/mL, patients with a PSA level above 4 ng/mL undergoing prostate MRI, and the mean number of days between PSA test and subsequent MRI among patients with a PSA result above 4 ng/mL are reported in Table 3. While the rates of prostate MRI remained below 1% in all age, race, and ethnicity groups (Tables 2 and 3), rates of receiving prostate MRI increased over time for each racial and ethnic group (eg, Hispanic: 2011, 3 [0.03%] patients vs 2017, 131 [0.3%] patients) (Table 3). Among patients with PSA levels above 4 ng/mL, the rate of receiving prostate MRI within 180 days after the PSA test date was highest among White patients (2017: 383 [4.5%] White patients vs 59 [2.9%] Black patients) and the rate also increased over time for all groups (Hispanic patients: 2011, 3 [0.8%] vs 2017, 106 [3.1%]). In addition, the highest prostate MRI rates were observed among men aged 65 to 74 years and the lowest rates were observed among older populations (eg, 2017: ages 65-74 years, 458 [0.6%] patients vs ≥85 years, 11 [0.2%] patients) (Table 2).

**Table 3.** Annual Frequencies for PSA, Elevated PSA, and Prostate MRI Examinations Performed Within 180 Days After a PSA Test Stratified by Age, Race, and Ethnicity

Year	Race/Ethnicity	Patients, No. (%)				Mean time between high PSA result and MRI, d^a^
		Receiving a PSA test	Undergoing MRI ≤180 d	With high PSA results^a^	Undergoing MRI ≤180 d after high PSA result^a^	
2011	White	57 602 (67.3)	58 (0.1)	2632 (4.6)	37 (1.4)	82 (43.8)
	Black	8671 (10.1)	4 (0.1)	506 (5.8)	4 (0.8)	52 (49.1)
	Hispanic	8693 (10.2)	3 (0.03)	383 (4.4)	3 (0.8)	101 (39.5)
	Asian	2761 (3.2)	1 (0.04)	85 (3.1)	1 (1.2)	31 (NA)
	Other^b^	7862 (9.2)	8 (0.1)	403 (5.1)	4 (1.0)	83 (26.2)
2012	White	49 802 (65.4)	64 (0.1)	2649 (5.3)	48 (1.8)	90 (47.7)
	Black	7791 (10.2)	11 (0.1)	490 (6.3)	10 (2.0)	62 (38.7)
	Hispanic	7851 (10.3)	8 (0.1)	381 (4.9)	5 (1.3)	95 (19.7)
	Asian	2983 (3.9)	2 (0.07)	137 (4.6)	2 (1.5)	91 (56.6)
	Other^b^	7719 (10.1)	9 (0.1)	453 (5.9)	5 (1.1)	85 (51.5)
2013	White	56 173 (63.1)	79 (0.1)	3109 (5.5)	59 (1.9)	89 (46.0)
	Black	9170 (10.3)	16 (0.1)	678 (7.4)	9 (1.3)	88 (32.1)
	Hispanic	9749 (11.0)	11 (0.1)	560 (5.7)	6 (1.1)	61 (45.7)
	Asian	3492 (3.9)	2 (0.06)	177 (5.1)	1 (0.6)	129 (NA)
	Other^b^	10 444 (11.7)	12 (0.1)	646 (6.2)	9 (1.4)	93 (58.5)
2014	White	46 704 (60.0)	73 (0.2)	2731 (5.9)	53 (1.9)	70 (45.8)
	Black	6995 (9.0)	11 (0.2)	511 (7.3)	7 (1.4)	87 (32.7)
	Hispanic	10 053 (12.9)	13 (0.1)	608 (6.1)	12 (2.0)	60 (49.3)
	Asian	3254 (4.2)	2 (0.1)	190 (5.8)	1 (0.5)	133 (NA)
	Other^b^	10 780 (13.9)	22 (0.2)	701 (6.5)	19 (2.7)	65 (50.5)
2015	White	57 197 (57.0)	148 (0.3)	3405 (6.0)	113 (3.3)	74 (44.3)
	Black	9309 (9.3)	20 (0.2)	678 (7.3)	18 (2.7)	74 (39.5)
	Hispanic	14 634 (14.6)	26 (0.2)	900 (6.2)	16 (1.8)	73 (49.0)
	Asian	4610 (4.6)	8 (0.2)	258 (5.6)	6 (2.3)	67 (34.9)
	Other^b^	14 601 (14.5)	45 (0.3)	950 (6.5)	37 (3.9)	71 (43.8)
2016	White	72 270 (55.3)	227 (0.3)	4407 (6.1)	180 (4.1)	72 (43.4)
	Black	13 799 (10.6)	55 (0.4)	1175 (8.5)	49 (4.2)	85 (44.8)
	Hispanic	17 104 (13.1)	34 (0.2)	1016 (5.9)	24 (2.4)	73 (38.4)
	Asian	5675 (4.3)	15 (0.3)	264 (4.7)	8 (3.0)	64 (43.5)
	Other^b^	21 876 (16.7)	74 (0.3)	1506 (6.9)	60 (4.0)	74 (44.0)
2017	White	115 466 (49.1)	473 (0.4)	8593 (7.4)	383 (4.5)	62 (44.0)
	Black	20 200 (8.6)	79 (0.4)	2016 (10.0)	59 (2.9)	71 (41.5)
	Hispanic	39 872 (17.0)	131 (0.3)	3430 (8.6)	106 (3.1)	58 (41.7)
	Asian	8575 (3.6)	29 (0.3)	611 (7.1)	25 (4.1)	70 (39.9)
	Other^b^	51 072 (21.7)	195 (0.4)	4261 (8.3)	145 (3.4)	51 (39.9)

^a^ High PSA results were considered levels 4 ng/mL or above.

^b^ Responses marking race and ethnicity as unknown or missing responses were combined into a single other category.

Bivariable regression results and *P* values are reported in eTable 2 and eTable 3 in the Supplement. Compared with White patients, Black and other patients with PSA results above 4 ng/mL and 10 ng/mL were significantly less likely to receive a prostate MRI within 180 days. Hispanic patients with PSA results above 10 ng/mL were also significantly less likely to undergo prostate MRI within 180 days after their PSA test date relative to White patients.

The odds ratios (ORs) from unadjusted bivariable regression models and from adjusted logistic regression models are reported in eTable 4 in the Supplement. The unadjusted and adjusted ORs for separate regression analyses for each age group and race/ethnicity are reported in eTable 5 and eTable 6 in the Supplement. The results show that patients who have Medicare are less likely to undergo prostate MRI within 180 days after their elevated PSA test date for all 3 PSA thresholds compared with patients with commercial insurance (eg, PSA levels >4 ng/mL: OR, 0.67; 95% CI, 0.52-0.87). Furthermore, patients with HMO insurance plan are less likely to receive prostate MRI within 180 days relative to patients with other insurance plan types (OR, 0.69; 95% CI, 0.55-0.86).

**Figure 1.**
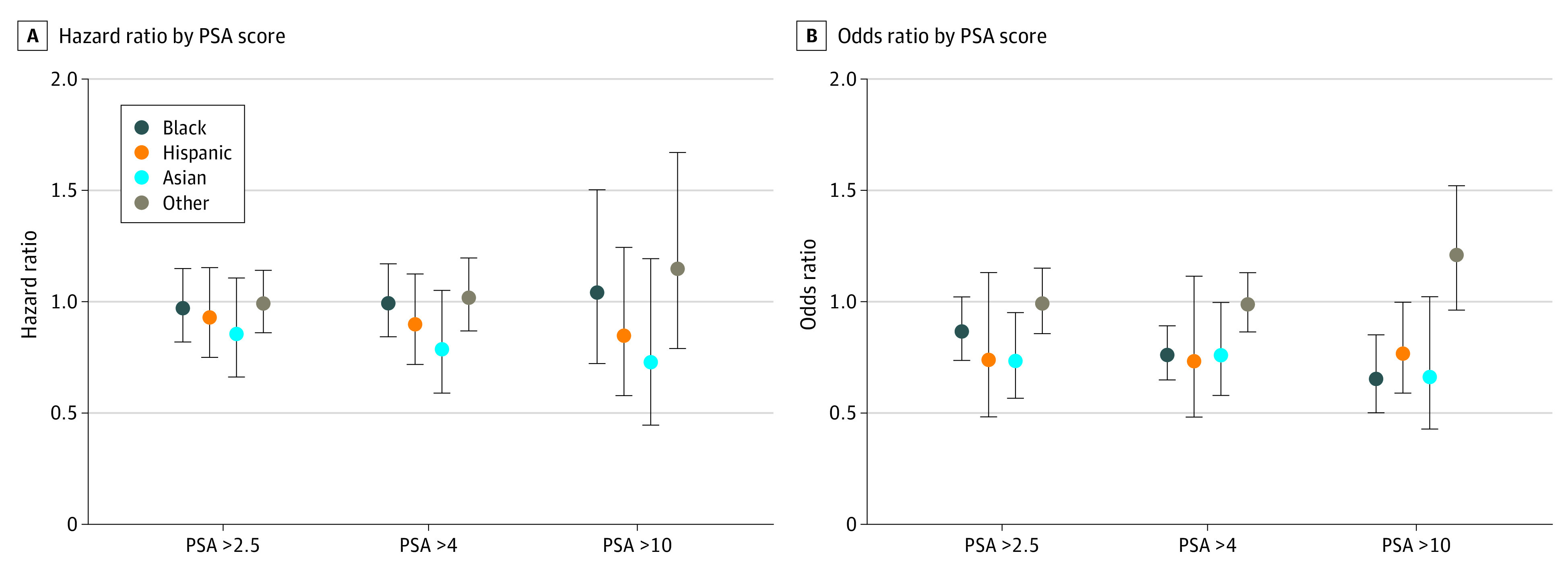
Multivariable Regression Results for Different PSA Levels PSA indicates prostate-specific antigen.

Figure 1 presents the adjusted ORs from multivariable logistic regression models separately for each race and ethnicity (with White patients used as the reference category). Compared with White patients, Black, Hispanic, and Asian patients were consistently less likely to receive prostate MRIs within 180 days across all 3 PSA thresholds. Specifically, Black patients with a PSA above 4 ng/mL were 24.1% less likely to undergo prostate MRI within 180 days after their PSA test date (OR, 0.76; 95% CI, 0.65-0.89), and those with a PSA level above 10 ng/mL were 35.0% less likely to undergo MRI (OR, 0.65; 95% CI, 0.50-0.85). Hispanic patients with a PSA above 10 ng/mL were 23.4% less likely to undergo prostate MRI compared with White patients (OR, 0.77; 95% CI, 0.59-0.99). Asian patients with PSA levels above 2.5 ng/mL and 4 ng/mL were 26.7% (OR, 0.73; 95% CI, 0.57-0.95) and 24.1% (OR, 0.76; 95% CI, 0.58-0.99) less likely to receive prostate MRI compared with White patients, respectively.

**Figure 2.**
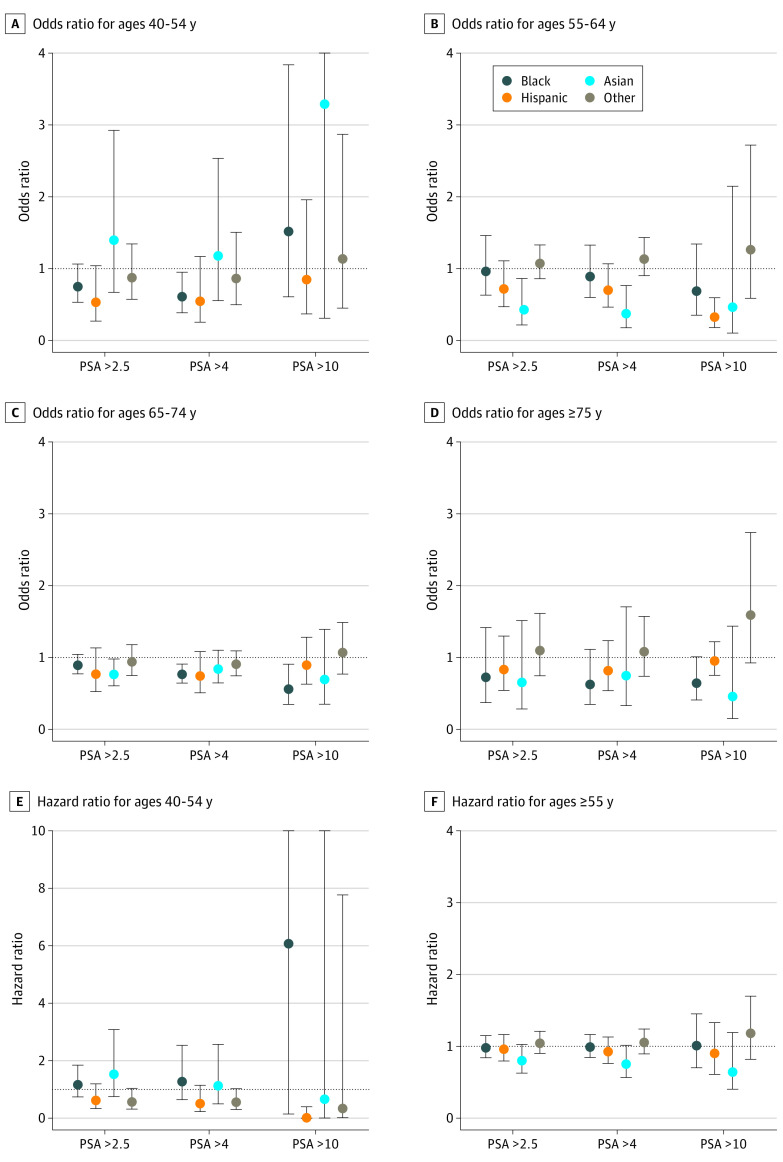
Multivariable Regression Results for Different Age Groups PSA indicates prostate-specific antigen.

The ORs for separate regression analyses for each age group and race and ethnicity are displayed in Figure 2. Black patients between 40 and 54 years of age and with a PSA result above 4 ng/mL were 39.8% less likely (OR, 0.60; 95% CI, 0.38-0.95) to undergo prostate MRI than White patients. Among patients ages 65 to 74 years, Black patients with PSA results above 4 ng/mL and 10 ng/mL were 23.6% (OR, 0.76; 95% CI, 0.64-0.91) and 43.9% (OR, 0.56; 95% CI, 0.35-0.91) less likely to receive prostate MRI compared with White patients, respectively. Asian patients between ages 55 and 64 years with a PSA result above 2.5 ng/mL and 4 ng/mL were 57.3% (OR, 0.43; 95% CI, 0.21-0.86) and 62.9% (OR, 0.37; 95% CI, 0.18-0.77) less likely to receive a subsequent prostate MRI than White patients. Finally, compared with White patients, Hispanic patients between ages 55 and 64 years with a PSA result above 10 ng/mL were 67.6% less likely to receive a subsequent prostate MRI (OR, 0.32; 95% CI, 0.18-0.56). The estimates for other age groups and PSA levels were not statistically significant.

Figures 1 and 2 present estimated coefficients from adjusted Cox regression models separately for race and ethnicity and each age group (ie, 40 to 54 years and ≥55 years). Among patients with an elevated PSA test result (PSA above 4 ng/mL), no statistically significant racial/ethnic disparities were observed in the number of days between PSA test and subsequent prostate MRI.

Finally, eTable 7 in the Supplement reports annual age-stratified frequencies and percentages of patients with PSA tests, patients with a subsequent MRI, patients with PSA levels above 4 ng/mL, patients with a PSA result above 4 ng/mL undergoing prostate MRI, and the mean number of days between PSA and subsequent MRI among patients with PSA results above 4 ng/mL for each of the 4 census regions (the West region, the South region, the Midwest region, and the Northeast region). Similarly, eTable 8 in the Supplement reports annual frequencies and percentages stratified by race and ethnicity of patients with PSA tests, patients undergoing prostate MRI, patients with a PSA result above 4 ng/mL, patients with a PSA result above 4 ng/mL undergoing prostate MRI, and the mean number of days between PSA test and subsequent MRI among patients with a PSA result above 4 ng/mL for each of the 4 regions. While rates of prostate MRI remained below 1% for all 4 regions, the highest rates were observed in the Northeast region across most years and racial and ethnic populations.

## Discussion

Using a large administrative data set that included both health insurance claims and laboratory results, we observed evidence of significant racial and ethnic disparities in the use of prostate MRI after PSA testing that widens with higher PSA results. Compared with White patients, Black, Hispanic, and Asian patients were significantly less likely to undergo prostate MRI within 180 days after an elevated PSA result, with significantly lower rates of prostate MRI at PSA thresholds of either 2.5, 4, or 10 ng/mL; this racial and ethnic disparity in the use of prostate MRI was most pronounced for the 4 ng/mL PSA threshold. Additionally, these disparities were observed across all age groups, but were greatest among patients between ages 55 and 64 years and in the Medicare-eligible population of patients ages 65 to 74 years with a PSA result above 10 ng/mL. These large racial and ethnic disparities highlight the need for additional research to better understand and mitigate clinical decision-making biases and other potential sources of these disparities, such as physician characteristics or biases in the health care system.^30,31^

Interestingly, the racial and ethnic disparities observed were insignificant among patients over 75 years of age—a population for which the US Preventive Services Task Force recommends against screening for prostate cancer.^32^ This indicates the important role that clearly defined guidelines can play in addressing racial and ethnic disparities in care. For the age groups for which prostate cancer screening is recommended, clearer guidelines are still needed for the optimal use of prostate MRI.

The observed annual increase in PSA tests is largely explained by substantial annual increases in Medicare Advantage enrollment in the data set during the study period. More concerning is the increasing percentage of PSA results above 4 ng/mL. Others have found that the US Preventative Services Task Force rating of D in 2012 led to a statistically significant and persistent decline in PSA screening in the primary care setting,^33^ which could account for a higher share of PSA tests above 4 ng/mL because PSA testing would be used more selectively—for example, only on patients with higher risk (family history or abnormal rectal exam).

In addition to a lack of clear guidelines on the use of prostate MRI following a PSA test, both unconscious and conscious biases may play a role in this health care inequity.^34^ For example, prior research has shown that physicians are less likely to discuss treatment options and potential side effects with Black vs White patients.^35^ Further research is needed to assess the role of these decision-making biases among physicians relative to other potential sources in the health care system for the observed racial and ethnic disparities in the use of prostate MRI, as well as examining whether these disparities extend to the use of prostate biopsy.

### Limitations

This study had several limitations. Although the analyzed data set covers all 50 states, the data came from a single source, which may limit the generalizability of our results to other insurance populations. Additionally, observations of our prostate MRI outcome variable may be missing because of patients dying or switching insurance companies, which could bias our results. The data set also did not contain information on physician or other patient characteristics (eg, general health status, family history with prostate cancer or other cancers, employment) that are important covariates for future investigators to consider. Additionally, while descriptive statistics do not indicate a significant change in racial and ethnic disparities in prostate cancer screening over time, more rigorous analyses are necessary to better estimate trends in prostate care. Finally, our results describe associations and should not be interpreted as causal.

## Conclusions

Significant racial and ethnic disparities exist in the use of prostate MRI following elevated PSA test results, and these disparities widen with higher PSA results. These disparities mirror those for other aspects of prostate cancer care^4^ and may contribute to known differences among racial/ethnic populations in prostate cancer outcomes.^36,37^ Policy efforts should be directed at eliminating these racial and ethnic disparities. Of note, clear guidelines on the use of prostate MRI in prostate cancer detection^38^ may help to standardize the evaluation for prostate cancer across all patients.

## Notes

Supplement. **eTable 1.** Insurance Claims Codes Used in the Analysis **eTable 2.** Bivariate Models and Models Stratified by PSA Level **eTable 3.** Model of Undergoing a Prostate MRI After an Elevated PSA on Race/Ethnicity Stratified by Age Groups **eTable 4.** Odds Ratios for Bivariate and Multivariable Regression Models of Undergoing a Prostate MRI After an Elevated PSA **eTable 5.** Odds Ratios for Bivariate and Multivariable Regression Models for Different PSA Levels **eTable 6.** Odds Ratios for Bivariate and Multivariable Regression Models Stratified by Age Groups **eTable 7.** Annual Frequency of PSA Testing and Subsequent MRI by Regions **eTable 8.** Race-Stratified Frequencies for PSA, Elevated PSA, and Prostate MRI Examinations Performed Within 180 Days After a PSA Test by Regions
