# Supplementary material for: Racial and Ethnic Disparities in the Use of Prostate Magnetic Resonance Imaging Following an Elevated Prostate-Specific Antigen Test
Source: JAMA Netw Open. 2021 Nov 8;4(11):e2132388. doi: 10.1001/jamanetworkopen.2021.32388 (PMC8576586; doi:10.1001/jamanetworkopen.2021.32388)
Supplement: Supplement. — eTable 1. Insurance Claims Codes Used in the Analysis eTable 2. Bivariate Models and Models Stratified by PSA Level eTable 3. Model of Undergoing a Prostate MRI After an Elevated PSA on Race/Ethnicity Stratified by Age Groups eTable 4. Odds Ratios for Bivariate and Multivariable Regression Models of Undergoing a Prostate MRI After an Elevated PSA eTable 5. Odds Ratios for Bivariate and Multivariable Regression Models for Different PSA Levels eTable 6. Odds Ratios for Bivariate and Multivariable Regression Models Stratified by Age Groups eTable 7. Annual Frequency of PSA Testing and Subsequent MRI by Regions eTable 8. Race-Stratified Frequencies for PSA, Elevated PSA, and Prostate MRI Examinations Performed Within 180 Days After a PSA Test by Regions [file jamanetwopen-e2132388-s001.pdf]

Supplemental Online Content

Abashidze N, Stecher C, Rosenkrantz AB, Duszak R Jr, Hughes DR. Racial and ethnic disparities in the use of prostate magnetic resonance imaging following an elevated prostate-specific antigen test. *JAMA Netw Open.* 2021;4(11):e2132388. doi:10.1001/jamanetworkopen.2021.32388

eTable 1. Insurance Claims Codes Used in the Analysis

eTable 2. Bivariate Models and Models Stratified by PSA Level

eTable 3. Model of Undergoing a Prostate MRI After an Elevated PSA on Race/Ethnicity Stratified by Age Groups

eTable 4. Odds Ratios for Bivariate and Multivariable Regression Models of Undergoing a Prostate MRI After an Elevated PSA

eTable 5. Odds Ratios for Bivariate and Multivariable Regression Models for Different PSA Levels

eTable 6. Odds Ratios for Bivariate and Multivariable Regression Models Stratified by Age Groups

eTable 7. Annual Frequency of PSA Testing and Subsequent MRI by Regions

eTable 8. Race-Stratified Frequencies for PSA, Elevated PSA, and Prostate MRI Examinations Performed Within 180 Days After a PSA Test by Regions

This supplemental material has been provided by the authors to give readers additional information about their work.

© 2021 Abashidze N et al. *JAMA Network Open.*

eTable 1. Insurance claims codes used in the analysis.

CPT	ICD-9	ICD-10
55700; 72195; 72196; 72197;	185; 60000; 79093; 2334; 60010; 6011; V1046; 60001; 6029; 60020; 60090; 6028; 2222; 2365; 6003; 60091; 60011; 6023; 6010; 6020; V1642; 6012; 6021; 6019;	C61; N400; R972; R9720; D075; N402; N411; Z8546; N401; N429; D4289; D291; D400; D4283; N403; N423; N410; N420; Z8042; N412; N421; N419;

Note: CPT: Current Procedural Terminology; ICD: International Classification of Diseases.

© 2021 Abashidze N et al. *JAMA Network Open.*

eTable 2. Bivariate models and models stratified by PSA level

				PSA>2.5	PSA>4	PSA>10
	Regression Coefficient (P-value)	Regression Coefficient (P-value)	Regression Coefficient (P-value)	Regression Coefficient (P-value)	Regression Coefficient (P-value)	Regression Coefficient (P-value)
	(1)	(2)	(3)	(4)	(5)	(6)
PSA>2.5	3.605					
	(0.0001)					
PSA>4		3.932				
		(0.0001)				
PSA>10			3.310			
			(0.0001)			
Black				-0.093	-0.214	-0.359
				(0.324)	(0.029)	(0.012)
Hispanic				-0.276	0.240	-0.281
				(0.283)	(0.258)	(0.076)
Asian				-0.211	0.247	-0.279
				(0.325)	(0.192)	(0.281)
Other				0.053	0.383	0.238
				(0.488)	(0.070)	(0.029)
Pseudo R2	0.184	0.230	0.076	0.001	0.002	0.004
Number of obs.	794,809	794,809	794,809	105,141	51,500	8,806

Note: This table presents regression coefficients from logistic models predicting the likelihood of undergoing a prostate MRI within 180 days of receiving an elevated PSA test result.

© 2021 Abashidze N et al. *JAMA Network Open.*

eTable 3. Model of undergoing a prostate MRI after an elevated PSA on race/ethnicity stratified by age groups

	Age [40; 54]			Age [55; 64]			Age [65; 74]			Age 75+		
	PSA>2.5	PSA>4	PSA>10	PSA>2.5	PSA>4	PSA>10	PSA>2.5	PSA>4	PSA>10	PSA>2.5	PSA>4	PSA>10
	(1)	(2)	(3)	(4)	(5)	(6)	(7)	(8)	(9)	(10)	(11)	(12)
Black	-0.317	-0.494	0.218	-0.083	-0.181	-0.472	-0.058	-0.219	-0.595	-0.085	-0.212	-0.232
	(0.091)	(0.040)	(0.657)	(0.695)	(0.354)	(0.162)	(0.558)	(0.054)	(0.010)	(0.787)	(0.441)	(0.326)
Hispanic	-0.507	-0.476	-0.115	-0.305	-0.332	-1.049	-0.253	-0.280	-0.153	-0.030	-0.021	0.032
	(0.124)	(0.208)	(0.802)	(0.152)	(0.105)	(0.0001)	(0.373)	(0.328)	(0.500)	(0.933)	(0.953)	(0.881)
Asian	0.410	0.333	1.451	-0.750	-0.937	-0.661	-0.174	-0.139	-0.324	-0.246	-0.127	-0.449
	(0.292)	(0.365)	(0.104)	(0.040)	(0.012)	(0.371)	(0.395)	(0.450)	(0.345)	(0.626)	(0.797)	(0.450)
Other	-0.027	-0.038	0.113	0.148	0.180	0.168	-0.028	-0.060	0.085	0.111	0.093	0.559
	(0.900)	(0.894)	(0.847)	(0.179)	(0.097)	(0.604)	(0.804)	(0.528)	(0.612)	(0.583)	(0.645)	(0.066)
Pseudo R2	0.004	0.006	0.012	0.002	0.003	0.014	0.001	0.001	0.006	0.000	0.001	0.005
Number of obs.	11,210	3,353	437	28,417	11,425	1,513	39,826	21,044	3,122	25,688	15,678	3,734

Note: This table presents regression coefficients from logistic models predicting the likelihood of undergoing a prostate MRI within 180 days of receiving an elevated PSA test result; P-values are reported in parentheses.

© 2021 Abashidze N et al. *JAMA Network Open.*

eTable 4. Odds-Ratios for bivariate and multivariable regression models of undergoing a prostate MRI after an elevated PSA.

	Bivariate Model (N=794,809)		Multivariable Model (N=794,809)		Bivariate Model (N=794,809)		Multivariable Model (N=794,809)		Bivariate Model (N=794,809)		Multivariable Model (N=794,809)	
Variable	OR (95% CI)	P-value	OR (95% CI)	P-value	OR (95% CI)	P-value	OR (95% CI)	P-value	OR (95% CI)	P-value	OR (95% CI)	P-value
PSA>2.5	36.77 (29.032;46.571)	<0.001	34.603 (26.214;45.675)	<0.001								
PSA>4					51.021 (42.802;60.819)	<0.001	52.88 (42.141;66.354)	<0.001				
PSA>10									27.392 (24.151;31.068)	<0.001	23.887 (19.901; 28.672)	<0.001
Black			0.918 (0.786; 1.073)	0.283			0.813 (0.697; 0.949)	0.009			0.849 (0.72; 1.001)	0.051
Hispanic			0.754 (0.535; 1.062)	0.107			0.74 (0.522; 1.049)	0.091			0.773 (0.533; 1.121)	0.175
Asian			0.741 (0.569; 0.964)	0.026			0.767 (0.567; 1.037)	0.085			0.714 (0.555; 0.919)	0.009
Other			1.005 (0.862; 1.171)	0.949			0.973 (0.833; 1.137)	0.734			0.993 (0.852; 1.158)	0.931
Age [55; 64]			1.51 (1.291; 1.764)	<0.001			1.549 (1.321; 1.815)	<0.001			3.136 (2.724; 3.611)	<0.001
Age [65; 74]			1.866 (1.495; 2.33)	<0.001			1.766 (1.405; 2.218)	<0.001			5.32 (4.292; 6.594)	<0.001
Age [75; 84]			1.316 (0.992; 1.745)	0.057			1.097 (0.819; 1.469)	0.537			3.683 (2.763; 4.908)	<0.001
Age 85+			0.431 (0.317; 0.586)	<0.001			0.304 (0.215; 0.43)	<0.001			0.901 (0.635; 1.28)	0.562
HMO			0.712 (0.609; 0.833)	<0.001			0.697 (0.596; 0.815)	<0.001			0.691 (0.595; 0.803)	<0.001
Medicare			0.719 (0.566; 0.913)	0.007			0.706 (0.561;0.889)	0.003			0.686 (0.535; 0.88)	0.003
Pseudo R2	0.184		0.216		0.23		0.267		0.076		0.126	

Note: This table presents exponentiated regression coefficients (i.e. odds ratios) from logistic models predicting the likelihood of undergoing a prostate MRI within 180 days of receiving an elevated PSA test result. Multivariable models control for year of the PSA test and patients' state of residence.

© 2021 Abashidze N et al. *JAMA Network Open.*

eTable 5. Odds-Ratios for bivariate and multivariable regression models for different PSA levels.

	PSA>2.5					PSA>4					PSA>10			
	Bivariate Model (N=105,141)		Multivariable Model (N=105,141)			Bivariate Model (N=51,500)		Multivariable Model (N=51,500)			Bivariate Model (N=8,806)		Multivariable Model (N=8,806)	
Variable	OR (95% CI)	P-value	OR (95% CI)	P-value		OR (95% CI)	P-value	OR (95% CI)	P-value		OR (95% CI)	P-value	OR (95% CI)	P-value
Black	0.911 (0.757; 1.096)	0.324	0.866 (0.735; 1.021)	0.087		0.808 (0.667; 0.978)	0.029	0.759 (0.648; 0.891)	0.001		0.699 (0.529; 0.923)	0.012	0.65 (0.5; 0.85)	0.002
Hispanic	0.759 (0.458; 1.256)	0.283	0.738 (0.482; 1.13)	0.162		0.739 (0.446; 1.224)	0.24	0.732 (0.48; 1.114)	0.145		0.755 (0.553; 1.03)	0.076	0.766 (0.588; 0.997)	0.047
Asian	0.81 (0.532; 1.233)	0.325	0.733 (0.565; 0.95)	0.019		0.801 (0.549; 1.166)	0.247	0.758 (0.578; 0.994)	0.046		0.757 (0.456; 1.256)	0.281	0.66 (0.427; 1.022)	0.062
Other	1.054 (0.908; 1.225)	0.488	0.992 (0.855; 1.15)	0.914		1.063 (0.927; 1.219)	0.383	0.987 (0.863; 1.13)	0.854		1.269 (1.025; 1.572)	0.029	1.209 (0.962; 1.521)	0.104
Age [55; 64]			1.138 (0.979; 1.322)	0.092				0.901 (0.776; 1.045)	0.168				0.98 (0.62; 1.55)	0.933
Age [65; 74]			1.304 (1.075; 1.582)	0.007				0.912 (0.757; 1.098)	0.329				0.942 (0.63; 1.407)	0.769
Age [75; 84]			0.849 (0.666; 1.084)	0.189				0.493 (0.38; 0.641)	<0.001				0.481 (0.303; 0.764)	0.002
Age 85+			0.257 (0.168; 0.395)	<0.001				0.138 (0.089; 0.214)	<0.001				0.153 (0.078; 0.301)	<0.001
HMO			0.698 (0.578; 0.844)	<0.001				0.687 (0.547; 0.862)	0.001				0.722 (0.579; 0.901)	0.004
Medicare			0.71 (0.562; 0.897)	0.004				0.676 (0.523; 0.874)	0.003				0.752 (0.536; 1.055)	0.099
Pseudo R2	0.001		0.044			0.002		0.061			0.004		0.086	

Note: This table presents exponentiated regression coefficients (i.e. odds ratios) from logistic models predicting the likelihood of undergoing a prostate MRI within 180 days of receiving an elevated PSA test result. Multivariable models control for year of the PSA test and patients' state of residence.

© 2021 Abashidze N et al. *JAMA Network Open.*

eTable 6. Odds-Ratios for bivariate and multivariable regression models stratified by age groups.

Panel A: Age group [40; 54]												
	PSA>2.5				PSA>4				PSA>10			
	Bivariate Model (N=11,210)		Multivariable Model (N=11,210)		Bivariate Model (N=3,353)		Multivariable Model (N=3,353)		Bivariate Model (N=437)		Multivariable Model (N=437)	
Variable	OR (95% CI)	P-value	OR (95% CI)	P-value	OR (95% CI)	P-value	OR (95% CI)	P-value	OR (95% CI)	P-value	OR (95% CI)	P-value
Black	0.728 (0.504; 1.052)	0.091	0.749 (0.527; 1.064)	0.106	0.61 (0.381; 0.979)	0.04	0.602 (0.383; 0.947)	0.028	1.244 (0.474; 3.265)	0.657	1.517 (0.6; 3.838)	0.379
Hispanic	0.602 (0.316; 1.15)	0.124	0.53 (0.27; 1.04)	0.065	0.621 (0.296; 1.304)	0.208	0.543 (0.253; 1.167)	0.118	0.891 (0.362; 2.197)	0.802	0.845 (0.365; 1.957)	0.694
Asian	1.507 (0.703; 3.233)	0.292	1.395 (0.665; 2.925)	0.378	1.395 (0.679; 2.868)	0.365	1.182 (0.551; 2.534)	0.667	4.265 (0.743; 24.474)	0.104	3.286 (0.309; 34.982)	0.324
Other	0.973 (0.636; 1.489)	0.9	0.874 (0.569; 1.343)	0.54	0.963 (0.55; 1.686)	0.894	0.861 (0.493; 1.503)	0.598	1.12 (0.356; 3.525)	0.847	1.131 (0.446; 2.867)	0.795
HMO			1.17 (0.696; 1.968)	0.553			1.134 (0.61; 2.105)	0.692			1.906 (0.33; 10.997)	0.471
Medicare			0.814 (0.473; 1.4)	0.457			0.954 (0.513; 1.776)	0.883			1.276 (0.294; 5.534)	0.745
Pseudo R2	0.004		0.051		0.006		0.071		0.012		0.184	
Panel B: Age group [55; 64]												
	Bivariate Model (N=28,417)		Multivariable Model (N=28,417)		Bivariate Model (N=11,425)		Multivariable Model (N=11,425)		Bivariate Model (N=1,513)		Multivariable Model (N=1,513)	
Black	0.921 (0.608; 1.393)	0.695	0.958 (0.628; 1.461)	0.841	0.835 (0.57; 1.223)	0.354	0.888 (0.595; 1.326)	0.563	0.624 (0.322; 1.209)	0.162	0.686 (0.351; 1.342)	0.271
Hispanic	0.737 (0.486; 1.119)	0.152	0.718 (0.465; 1.108)	0.134	0.718 (0.48; 1.072)	0.105	0.7 (0.46; 1.064)	0.095	0.35 (0.197; 0.621)	<0.001	0.324 (0.177; 0.594)	<0.001
Asian	0.472 (0.231; 0.967)	0.04	0.427 (0.212; 0.86)	0.017	0.392 (0.189; 0.813)	0.012	0.371 (0.179; 0.767)	0.007	0.516 (0.121; 2.2)	0.371	0.463 (0.1; 2.144)	0.325
Other	1.159 (0.935; 1.438)	0.179	1.067 (0.857; 1.329)	0.561	1.197 (0.968; 1.481)	0.097	1.134 (0.898; 1.431)	0.291	1.183 (0.627; 2.231)	0.604	1.262 (0.586; 2.719)	0.552
HMO			0.411 (0.279; 0.606)	<0.001			0.381 (0.234; 0.618)	0			0.652 (0.319; 1.331)	0.24
Medicare			0.728 (0.455; 1.164)	0.185			0.63 (0.41; 0.968)	0.035			0.533 (0.292; 0.974)	0.041
Pseudo R2	0.002		0.04		0.003		0.046		0.014		0.081	

© 2021 Abashidze N et al. *JAMA Network Open.*

Panel C: Age group [65; 74]												
	PSA>2.5				PSA>4				PSA>10			
	Bivariate Model (N=39,826)		Multivariable Model (N=39,826)		Bivariate Model (N=21,044)		Multivariable Model (N=21,044)		Bivariate Model (N=3,122)		Multivariable Model (N=3,122)	
Black	0.943<br>(0.776; 1.146)	0.558	0.897<br>(0.769; 1.046)	0.165	0.803<br>(0.643; 1.004)	0.054	0.764<br>(0.642; 0.911)	0.003	0.552<br>(0.35; 0.869)	0.01	0.561<br>(0.347; 0.907)	0.018
Hispanic	0.777<br>(0.445; 1.355)	0.373	0.772<br>(0.524; 1.136)	0.189	0.756<br>(0.431; 1.325)	0.328	0.744<br>(0.51; 1.086)	0.125	0.858<br>(0.55; 1.339)	0.5	0.897<br>(0.627; 1.284)	0.552
Asian	0.84<br>(0.563; 1.254)	0.395	0.769<br>(0.602; 0.983)	0.036	0.87<br>(0.607; 1.248)	0.45	0.841<br>(0.641; 1.103)	0.211	0.723<br>(0.369; 1.418)	0.345	0.699<br>(0.349; 1.399)	0.311
Other	0.972<br>(0.78; 1.213)	0.804	0.941<br>(0.751; 1.178)	0.595	0.942<br>(0.782; 1.134)	0.528	0.903<br>(0.744; 1.095)	0.298	1.089<br>(0.784; 1.512)	0.612	1.068<br>(0.766; 1.489)	0.698
HMO			0.775<br>(0.587; 1.024)	0.073			0.764<br>(0.555; 1.051)	0.098			0.671<br>(0.489; 0.92)	0.013
Medicare			0.782<br>(0.551; 1.11)	0.17			0.787<br>(0.518; 1.197)	0.263			1.01<br>(0.62; 1.645)	0.969
Pseudo R2	0.001		0.039		0.001		0.045		0.006		0.074	
Panel D: Age group 75+												
	Bivariate Model (N=25,688)		Multivariable Model (N=25,688)		Bivariate Model (N=15,678)		Multivariable Model (N=15,678)		Bivariate Model (N=3,734)		Multivariable Model (N=3,734)	
Black	0.918<br>(0.495; 1.703)	0.787	0.722<br>(0.368; 1.416)	0.343	0.809<br>(0.471; 1.388)	0.441	0.62<br>(0.347; 1.109)	0.107	0.793<br>(0.499; 1.26)	0.326	0.64<br>(0.407; 1.007)	0.054
Hispanic	0.971<br>(0.485; 1.942)	0.933	0.833<br>(0.536; 1.294)	0.415	0.979<br>(0.482; 1.989)	0.953	0.813<br>(0.534; 1.236)	0.332	1.033<br>(0.675; 1.58)	0.881	0.953<br>(0.747; 1.216)	0.7
Asian	0.782<br>(0.29; 2.106)	0.626	0.65<br>(0.28; 1.512)	0.317	0.881<br>(0.336; 2.312)	0.797	0.747<br>(0.327; 1.706)	0.489	0.638<br>(0.199; 2.045)	0.45	0.456<br>(0.145; 1.436)	0.18
Other	1.118<br>(0.751; 1.663)	0.583	1.093<br>(0.742; 1.61)	0.653	1.097<br>(0.739; 1.628)	0.645	1.076<br>(0.736; 1.573)	0.705	1.749<br>(0.964; 3.172)	0.066	1.592<br>(0.924; 2.743)	0.094
HMO			0.523<br>(0.322; 0.848)	0.009			0.502<br>(0.339; 0.744)	0.001			0.513<br>(0.235; 1.116)	0.092
Medicare			0.773<br>(0.378; 1.579)	0.48			0.846<br>(0.386; 1.855)	0.676			0.877<br>(0.314; 2.454)	0.803
Pseudo R2	0.0003		0.057		0.001		0.065		0.005		0.09	

© 2021 Abashidze N et al. *JAMA Network Open.*

Note: This table presents exponentiated regression coefficients (i.e. odds ratios) from logistic models predicting the likelihood of undergoing a prostate MRI within 180 days of receiving an elevated PSA test result. Multivariable models control for year of the PSA test and patients' state of residence.

© 2021 Abashidze N et al. *JAMA Network Open.*

eTable 7. Annual frequency of PSA testing and subsequent MRI by regions

	Year	Patients receiving a PSA test	Number undergoing MRI within 180 days (%)		Number with PSA>4 (%)		Number undergoing MRI within 180 days if PSA>4 (180 days) (%)		Mean days between PSA>4 and MRI
West Region	2011	14,206	8	0.06%	769	5%	8	1.04%	68
	2012	12,951	12	0.09%	770	6%	10	1.30%	81
	2013	16,396	7	0.04%	1,004	6%	3	0.30%	127
	2014	17,072	20	0.12%	1,162	7%	16	1.38%	72
	2015	21,426	25	0.12%	1,524	7%	20	1.31%	80
	2016	26,659	52	0.20%	1,793	7%	41	2.29%	84
	2017	48,412	140	0.29%	4,091	8%	114	2.79%	59
South Region	2011	56,792	45	0.08%	2,493	4%	29	1.16%	83
	2012	47,460	59	0.12%	2,268	5%	45	1.98%	89
	2013	54,617	67	0.12%	2,900	5%	44	1.52%	89
	2014	43,406	61	0.14%	2,288	5%	45	1.97%	66
	2015	58,902	137	0.23%	3,313	6%	100	3.02%	69
	2016	80,204	232	0.29%	4,757	6%	184	3.87%	75
	2017	149,119	522	0.35%	11,731	8%	410	3.50%	61
Midwest Region	2011	8,595	11	0.13%	471	5%	7	1.49%	83
	2012	7,724	4	0.05%	434	6%	3	0.69%	79
	2013	9,304	16	0.17%	573	6%	11	1.92%	104
	2014	8,756	12	0.14%	582	7%	9	1.55%	72
	2015	8,772	31	0.35%	502	6%	26	5.18%	79
	2016	10,855	32	0.29%	712	7%	27	3.79%	86
	2017	15,900	71	0.45%	1,132	7%	58	5.12%	68
Northeast Region	2011	5,996	10	0.17%	276	5%	5	1.81%	75
	2012	8,011	19	0.24%	636	8%	12	1.89%	81
	2013	8,711	30	0.34%	693	8%	26	3.75%	75
	2014	8,552	28	0.33%	709	8%	22	3.10%	76
	2015	11,251	54	0.48%	852	8%	44	5.16%	75
	2016	13,006	89	0.68%	1,106	9%	69	6.24%	62
	2017	21,754	174	0.80%	1,957	9%	136	6.95%	55

© 2021 Abashidze N et al. *JAMA Network Open.*

eTable 8. Race-stratified frequencies for PSA, elevated PSA, and prostate MRI examinations performed within 180 days after a PSA test by regions.

	Race/Ethnicity	Patients receiving a PSA test	Number undergoing MRI within 180 days (%)		Number with PSA>4 (%)		Number undergoing MRI within 180 days if PSA>4 (180 days) (%)		Mean days between PSA>4 and MRI
West Region	White	92,066	159	0.17%	6,397	7%	132	2.06%	70
	Black	3,080	5	0.16%	246	8%	5	2.03%	53
	Hispanic	22,903	31	0.14%	1,646	7%	22	1.34%	81
	Asian	8,838	10	0.11%	565	6%	8	1.42%	55
	Other	30,235	59	0.20%	2,261	7%	45	1.99%	64
South Region	White	266,258	594	0.22%	14,629	5%	445	3.04%	71
	Black	61,981	150	0.24%	4,692	8%	119	2.54%	79
	Hispanic	73,187	154	0.21%	4,628	6%	116	2.51%	63
	Asian	14,923	23	0.15%	678	5%	18	2.65%	68
	Other	74,151	202	0.27%	5,123	7%	159	3.10%	61
Midwest Region	White	51,455	128	0.25%	3,089	6%	105	3.40%	78
	Black	5,224	15	0.29%	505	10%	12	2.38%	86
	Hispanic	3,220	4	0.12%	177	5%	3	1.69%	82
	Asian	1,788	1	0.06%	85	5%	1	1.18%	28
	Other	8,219	29	0.35%	550	7%	20	3.64%	69
Northeast Region	White	45,435	241	0.53%	3,411	8%	191	5.60%	65
	Black	5,650	26	0.46%	611	11%	20	3.27%	63
	Hispanic	8,646	37	0.43%	827	10%	31	3.75%	53
	Asian	5,801	25	0.43%	394	7%	17	4.31%	85
	Other	11,749	75	0.64%	986	8%	55	5.58%	60

© 2021 Abashidze N et al. *JAMA Network Open.*
